# Social Information Processing Theory Indicators of Child Abuse Risk: Cultural Comparison of Mothers from Peru and the United States

**DOI:** 10.3390/children10030545

**Published:** 2023-03-13

**Authors:** Christina M. Rodriguez, Patricia Bárrig Jó, Enrique Gracia, Marisol Lila

**Affiliations:** 1 Department of Psychology, Old Dominion University, 250 Mills Godwin Building, Norfolk, VA 23529, USA; 2 Department of Psychology, Pontifical Catholic University of Peru, Av. Universitaria 1801, San Miguel, Lima 15088, Peru; 3 Department of Social Psychology, University of Valencia, Av. Blanco Ibáñez, 21, 46010 Valencia, Spain

**Keywords:** Social Information Processing theory, child maltreatment, physical abuse, psychological abuse, child abuse risk, cross-cultural

## Abstract

Much of the research conducted on social information processing (SIP) factors predictive of child abuse risk has been conducted in North America, raising questions about how applicable such models may be in other cultures. Based on the premise that the parents’ child abuse risk is affected by both risk and protective factors, the current study considered how specific SIP socio-cognitive risk factors (acceptability of parent–child aggression as a discipline approach; empathic ability; frustration tolerance) as well as social support satisfaction as a resource related to child abuse risk by comparing a sample of mothers in Peru (*n* = 102) with a sample of mothers in the U.S. (*n* = 180). Using multi-group regression analyses, the current investigation identified that lower empathy was more salient for the abuse risk of U.S. mothers relative to the salience of lower frustration tolerance for Peruvian mothers. Although effects were observed for the approval of parent-aggression for the child abuse risk of both samples, such approval did not appear to be related to the Peruvian mothers’ actual use of such tactics. When considered alongside the socio-cognitive risk factors, greater social support satisfaction did not significantly relate to child abuse risk for either sample. The findings are discussed in reference to future cross-cultural work that may need to better examine how factors may or may not be universal to craft more culturally informed child abuse prevention programs.

## 1. Introduction

Child protective services identify physical abuse when a parent intentionally employs physical force that incurs childhood injury and psychological abuse when a parent engages in practices that result in mental harm [1]. Although both physical abuse and psychological abuse are associated with a host of long-term psychological challenges [2,3], some meta-analyses have observed stronger effects from psychological abuse in the development of depression [4] and the maladaptive schemas characteristic of psychopathology in general [5]. Such findings underscore the need to consider both forms of parental maltreatment of children as critical adverse childhood experiences.

Despite abuse reporting laws, only a fraction of maltreatment is officially reported to child protective services [6,7], complicating the ability of researchers to solely rely on substantiated reports of abuse. Alternatively, child abuse risk is measured, serving as an estimate of a parent’s likelihood to engage in child maltreatment, by assessing the attitudes and behaviors predictive of subsequent referral for maltreatment [8,9]. The behavior most likely to lead to child maltreatment involves parents’ use of any form of parent–child aggression (PCA). When parents’ use of familiar forms of physical or emotional punishment (e.g., yelling, spanking) escalates in its intensity and/or frequency, child maltreatment is significantly more likely to transpire [10,11,12,13]. Specifically, abuse often begins with a parent’s aggressive response to child misbehavior, so that those who physically discipline are exponentially more likely to ultimately abuse including abuse that is officially substantiated [13,14]. Use of any form of physical PCA strongly overlaps with psychological aggression and emotional abuse [10,15], with spanking demonstrating negative effects comparable to other adverse childhood experiences [16]. In effect, PCA essentially operates along a continuum, with the familiar, milder forms (e.g., yelling, spanking) at one endpoint and extreme child abuse at the other endpoint [17,18]. Assessing the parents’ use of any PCA earlier on the continuum thus provides an estimate of a parent’s risk to abuse further along the continuum.

However, the vast majority of research about the predictors of PCA has emerged from North America, skewing the research conclusions that can be drawn more broadly [19]. This geographical focus raises questions regarding the applicability of the extant research findings on child abuse risk to other contexts. The current paper aimed to examine whether factors associated with PCA showed comparable effects—demonstrative of cross-cultural equivalence—specifically between a sample of Peruvian mothers and a sample of U.S. mothers that focused on the potential theoretical risk factors and the potential resource of social support.

One model of socio-cognitive risks that has been utilized toward understanding PCA is Social Information Processing (SIP) theory [20,21,22]. SIP theory has been applied to predict the PCA risk for parents of children of varying ages [20] including parents of toddlers (e.g., [22]), parents of preschoolers (e.g., [23]), and parents of school-age children (e.g., [24]). Although this research affirms the applicability of SIP across developmental periods, most research on SIP is dominated by work in the U.S. SIP theory proposes that before a parent is confronted with a discipline encounter, they carry several preconceived beliefs that will influence their likelihood of using PCA when conflict arises. Once a discipline situation arises, parents may misperceive the situation (Stage 1), formulate negative interpretations (Stage 2), and fail to adequately consider potentially mitigating information and discipline options (Stage 3), culminating in the implementation of PCA. One of the pre-existing schemas includes the belief and attitude that the use of PCA is an acceptable discipline approach. Additionally, a parent’s ability to empathize has been considered as a pre-existing schema [20,21], but the inability to empathize and adopt their child’s perspective could also be conceptualized as affecting Stage 3 processing [25,26] by interfering with a parent’s ability to incorporate mitigating information. Parents’ negative affect such as anger and frustration also potentially increase the salience of child misbehavior and evoke reactivity that can affect parents’ accuracy in their perceptions during Stage 1 processing [21,26,27].

Current research on pre-existing schemas supports that the acceptability of PCA use is associated with parents’ child abuse risk [22,28,29,30], a finding that has been observed worldwide [31] and across countries [32]. Parents’ limited empathic abilities have been identified in abusive [33] and suspected abusive mothers [34] as well as linked to elevated PCA risk [24,35]. Furthermore, parents who experience difficulties regulating anger and frustration are more over-reactive, increasing their child abuse risk [36,37], with evidence that poor frustration tolerance is particularly problematic [25,38].

Apart from such socio-cognitive risk factors, the research findings have documented that parents’ personal resources are linked to lower child abuse risk [39], consistent with a framework that considers both risks and protective factors. Social support, in particular, has been identified as one of the resources relevant for reduced parental child abuse risk [37,39,40,41], particularly for mothers [42]. However, the beneficial effect of social support has not been observed in some cultures such as in South Africa [43], suggesting that its effect may not be universal.

Because much of the research conducted on SIP processes has been performed in the U.S. (see [20] for review), its applicability elsewhere remains unclear, particularly in cultures that are less individualistic. Existing evidence supports the significance of culture on parenting beliefs and practices in general [44] and child abuse in particular [45]. Notably, the World Health Organization [46] documents high worldwide prevalence rates of child maltreatment. Because many research interpretations dominated by U.S. samples imply that the findings are generalizable and universal [47], more research is clearly needed outside North America to test such premises.

Although parents in the U.S. may endeavor to cultivate independence, more collectivistic cultures may approach parenting and discipline differently, emphasizing interdependence and mutuality [48]. Among Latin American parents, an emphasis on cooperation, respect, and expectations about contributing to the family is apparent [49]. In the South American context, our current understanding of processes contributing to child abuse risk in Peru remains relatively limited, although the prevalence rates for physical abuse exceed those observed in the U.S., with no clear data available on psychological abuse (see WHO interactive site, apps.who.int/violence-info/child-maltreatment). However, based on some nationwide surveys, substantial majorities of Peruvian children appear to have experienced both psychological and physical violence [50,51], with parental maltreatment identified as a strong predictor of peer violence at school [52]. A recent meta-regression of predictors of physical violence toward children across several low- and middle-income countries including Peru primarily focused on sociodemographic risk factors for child abuse risk [53].

Whereas PCA maintains high approval rates [54] and remains frequently implemented by parents in the U.S. [55], all forms of physical punishment of children were officially banned in Peru in 2015 [56]. Despite such prohibitions, a significant minority of Peruvian parents agreed with occasional physical discipline of children [56]. One of the few studies seeking to predict maternal use of physical PCA in Peru controlled for sociodemographic factors and identified a personal history of family violence as a significant predictor [57]. Given the more collectivistic, interdependent culture within Peru [58], social support could conceivably be more important for Peruvian parents, thereby serving as a stronger resource, and lowering their child abuse risk. A recent study of parents in Lima observed that, consistent with findings elsewhere, harsh parental punishment predicted the overall trauma victimization of children [59] and identified a relation between mothers’ overall resilience and reduced use of harsh punishment [60]. To date, no research has considered the applicability of socio-cognitive factors collectively as predictors of Peruvian parents’ child abuse risk nor evaluated both physical and psychological child abuse risk.

Given calls to consider the comparability of the findings observed in the U.S. to groups outside the U.S. [47,61], the current study contrasted a sample of Peruvian mothers with a sample of mothers in the U.S that focused on potential differences in the role of SIP factors in child abuse risk. The present study examined the potential relations of several SIP factors (PCA acceptability, frustration tolerance, empathic ability) as well as social support satisfaction as a potential resource, in relation to maternal child abuse risk (including the mothers’ actual physical and psychological PCA use), with the specific goal of testing whether these relations were comparable between mothers in both groups. Testing this collection of predictors simultaneously permits an examination of which processes were most salient per group beyond the variance attributable to the sociodemographic qualities that would differ between the countries.

## 2. Methods

### 2.1. Participants and Procedures

The current sample included two groups of mothers. The first group involved a community sample of 180 mothers in the U.S. enrolled in the “Following First Families” (Triple-F) study, a prospective longitudinal study tracking parent–child aggression risk in 203 families in the Southeast U.S. For the Triple-F study, mothers were recruited with flyers at local community health centers and hospital OB/GYN clinics; interested mothers contacted the research lab to schedule an appointment. For the present analysis, data were extracted from the third wave of the study when the focal child in the Triple-F study would have been 18 months old. Mothers provided written informed consent and all measures were delivered electronically on laptop computers. The university’s Institutional Review Board approved all study procedures for the Triple-F study.

Mothers at this time point were on average 27.6 years old (*SD* = 5.76). In terms of racial/ethnic characterization, mothers self-identified as: 48.9% White, 48.3% Black/African-American, 1.7% Native American, and 1.1% Asian; 3.3% of mothers also identified as Hispanic/Latina and 7.8% identified as biracial. Mothers’ highest level of education was reported as: 25.6% high school or less; 25.0% some college or vocational training; 21.1% college degree; and 28.3% > college degree. Regarding income, 48% of mothers reported an annual household income of <USD 40,000, with 39% reporting receipt of government public assistance.

The second group of mothers involved a community sample of 102 mothers residing in Lima, Peru, recruited through flyers posted at daycares, after school programs, and a church community outreach program. All mothers were required to have one child younger than eight years old who would be the focus of this study. Mothers contacted the researchers if interested in participating in scheduling a session. Mothers provided informed consent and completed measures anonymously, delivered electronically on laptop computers. The Institutional Review Board approved the study procedures for this data collection.

The Peruvian mothers were on average 33.5 years old (*SD* = 6.60). The mothers self-reported their race/ethnicity as: 77.5% Mestiza, 7.8% Amerindian, 4.9% Afroperuana, 3.9% European/Blanca, and 5.9% Other. Regarding the highest educational attainment based on the Peruvian educational system, 3.9% indicated primary school or less, 47.1% reported secondary education, and 52.9% indicated at least some college. Regarding income, 54.9% of mothers indicated an annual income between PEN 7000–9999, which would indicate below the average for Peruvians overall.

### 2.2. Measures

#### 2.2.1. Predictors

The *Physical Abuse Vignettes* [62] were utilized to estimate mothers’ acceptability of PCA. The measure presents eight vignettes of parents interacting with their child involving varying PCA intensity, from hitting a child to burning with a cigarette. Mothers were asked to indicate (1) whether they viewed the parental behavior as maltreatment (Yes/No, scored 1 and 0, respectively), summed across items for a definition total score; (2) how serious they considered the parental behavior on a 4-point scale (1 = least serious, 4 = most serious), summed across items for a severity total score; and (3) whether they would report the situation to child protection authorities (Yes/No, scored 1 and 0, respectively), summed across items for a reporting total score. On all three subscales, higher scores thus suggest less acceptability of PCA. Because the subscales involved different scales of measurement, the scores were standardized and combined into a total Vignette PCA Acceptability score separately by country for use in the primary analyses. Prior work using these vignettes provided convergent validity evidence with other measures of PCA acceptability [26]. Findings from the current study indicate acceptable internal consistency of the Vignette PCA Acceptability score for both the U.S. mothers (α = 0.81) and Peruvian mothers (α = 0.85).

The *Frustration Discomfort Scale* (FDS; [63]) includes seven items that indicate the respondent’s ability to tolerate frustration. Each item involves a 5-point Likert scale from 1 (strongly disagree) to 5 (strongly agree). Total scores can be computed by summing across items and oriented where higher scores denote poorer frustration tolerance. The FDS has demonstrated good psychometric properties including validity by distinguishing the clinical from the comparison samples [63]. The FDS scores in the current study also demonstrated good internal reliability for U.S. mothers (α = 0.90) and Peruvian mothers (α = 0.84).

The *Interpersonal Reactivity Index* (IRI; [64]) is a frequently used measure of empathic ability. The current investigation utilized responses from two subscales with seven items each: empathic concern and perspective taking (the ability to affectively sympathize with another and the ability to assume the perspective of another, respectively). Participants indicate how much each item characterizes them on a 5-point scale, from 1 (does not describe me well) to 5 (describes me very well). Items from both subscales were combined for an IRI total score wherein higher scores indicate greater empathic ability. The IRI demonstrates convergent validity [64]. In the current study, the IRI obtained acceptable internal consistency for the U.S. mothers (α = 0.82) and Peruvian mothers (α = 0.69).

The *Social Support Resources Index* (SSRI; [65]) was administered to measure mothers’ satisfaction with their social support network. Mothers were asked to report on the two strongest supporters who provided them with emotional and practical support and guidance. Mothers indicated how satisfied they were with each relationship using five items rated on a 5-point Likert scale from 1 (not satisfied) to 5 (very satisfied). Total social satisfaction scores were generated by summing across items for both supporters, with higher scores indicative of greater social satisfaction. Such scores have demonstrated validity with other measures of perceived support [65]. The SSRI demonstrated strong internal consistency for both samples in the current investigation (α = 0.94 for U.S. mothers and α = 0.92 for Peruvian mothers).

#### 2.2.2. Dependent Variables

The *Adult-Adolescent Parenting Inventory-2* (AAPI-2; [66]) is a measure of child abuse risk assessing parents’ beliefs and behaviors that characterize those engaged in child maltreatment. Mothers were asked to rate the 40 AAPI-2 items on a 5-point Likert scale, from 1 (strongly disagree) to 5 (strongly agree), which were summed for a total score oriented so that higher scores suggest greater child abuse risk. AAPI-2 items were those that distinguished maltreating from non-maltreating parents [67], with additional support for validity [68]. In the current investigation, the AAPI-2 total score demonstrated good reliability, with α = 0.91 for both U.S. and Peruvian mothers.

The *Parent–Child Conflict Tactics Scale* (CTSPC; [69]) is a frequently used measure to assess PCA and maltreatment worldwide [70]. Mothers were asked to report on how frequently they used 22 discipline approaches, with 13 items focused on physical assault and five items focused on psychological aggression. Items vary widely in PCA intensity (from spanking and yelling to scalding or calling the child names). CTSPC items are variably weighted categories based on reported frequency: 0, 1, or 2 times in the past year receive those values; 3–5 times receives a weight of 4; 6–10 times receives a weight of 8; 11–20 times receives a weight of 15; and more than 20 times receives a weight of 25. The current study combined the physical and psychological responses for a CTSPC Combined Assault score. The test authors provide evidence of construct and discriminant validity.

#### 2.2.3. Measure Translation Procedures

The AAPI-2 is available in Spanish and the IRI has previously been translated into Spanish [71]. The remaining measures were first translated by two independent native Spanish speakers. The full set of measures were then back-translated by another native Spanish speaker, with consensus across the team to address differences. Finally, the consensus set of translated measures was reviewed and adjusted as needed for the Peruvian content by a native Peruvian speaker.

### 2.3. Analytical Plan

Preliminary analyses of the group differences between samples, need for potential covariates, and bivariate analyses within country were first performed with SPSS 27.0, with a minimum *p* ≤ 0.05 adopted as the indicator of statistical significance. The preliminary independent samples *t*-test followed the conventional form:

$$t=\frac{{\bar{x}}_{1}-{\bar{x}}_{2}}{\sqrt{\frac{s^{2}}{N_{1}}+\frac{s^{2}}{N_{2}}}}$$

where the $\bar{x}$ signifies the mean; *s* signifies the standard deviation; and *N* signifies the sample size of the respective samples 1 and 2. Multi-group multiple regression analyses were then conducted utilizing Mplus 8.1 [72] with missing values accommodated using full-information maximum likelihood methods (FIML). All predictors were entered simultaneously regressed on both dependent variables simultaneously (AAPI-2 and CTSPC)—all in a single model. This approach reduces the number of statistical tests and provides strong controls to identify the potentially strongest predictors separately for either outcome measure while accounting for any potential covariation among predictors. Because the primary interest was in the potential differences in the path coefficients between the two samples of mothers, the model was fully identified and the model fit indices are thus uninformative. Significant path coefficients denote a significant relation between the predictor and the outcome measure in the fully controlled model. To identify potential significant differences in the relations between the groups, coefficients for a given path were constrained to be equal between samples, with significant differences between paths identified using Wald statistics. The Wald statistic followed the formula:

$$W=\frac{{\left(\hat{\theta }-\theta_{0}\right)}^{2}}{\mathrm{var}\left(\hat{\theta }\right)}$$

where *W* tests for significance in the difference of the Chi-square of the model with the maximum-likelihood estimation of the parameter value $\hat{\theta }\;$ derived from the sample data against the Chi-square of the model with a null hypothesized coefficient *θ*_0_, in which the path coefficients are constrained to be equal between groups. Significant Wald test statistics thus indicate that the given path coefficient significantly differs between groups.

## 3. Results

### 3.1. Preliminary Analyses

Initial analyses indicated that samples significantly differed in maternal age, *t* = 7.76, *p* < 0.001, likely reflecting that the U.S. sample involved first-time mothers because parental age is collinear with child age. In addition, maternal age was significantly related to the AAPI-2 for both the Peruvian and U.S. mothers. We also computed a socioeconomic status (SES) indicator based on the standardized values of both educational attainment and income, which were also collinear; because countries differ in such indicators, standardization was established separately per country. SES also demonstrated significant relations with the AAPI-2 as well as social support and IRI for U.S. mothers, and with AAPI-2 and FDS scores for Peruvian mothers. Because maternal age and SES were related to outcome measures, the multiple regression analyses utilized both as demographic covariates that were not substantially collinear.

Means and standard deviations across measures for each sample are shown in Table 1. Peruvian mothers were statistically significantly more likely to judge the vignette scenes as abusive, more serious, and worth reporting to authorities relative to the U.S. mothers. However, the U.S. mothers reported statistically significantly more empathy and social satisfaction than the Peruvian mothers; in addition, the U.S. mothers obtained statistically significantly lower abuse risk scores on the AAPI-2 and CTSPC than the Peruvian mothers. The samples did not statistically significantly differ from each other in terms of frustration tolerance.

Table 2 provides the results of the bivariate analyses by country. For Peruvian mothers, greater PCA acceptability was significantly related to greater abuse risk on only one of the two dependent variables (AAPI-2); in contrast, greater PCA acceptability was significantly related to higher abuse risk scores for both dependent variables for U.S. mothers. Low frustration tolerance was weakly related to greater child abuse risk on both measures for U.S. mothers but moderately related for Peruvian mothers on the AAPI-2. Greater empathy was not significantly related to lower abuse risk for Peruvian mothers but was associated with lower abuse risk on the AAPI-2 for U.S. mothers. Greater social satisfaction was significantly related to lower abuse risk on the AAPI-2 for Peruvian mothers, with no significant associations observed between low social support and greater abuse risk for U.S. mothers.

### 3.2. Multi-Group Multiple Regression Analyses

Results from the Mplus analysis are shown in Table 3. Notably, greater PCA acceptability significantly predicted higher abuse risk on the AAPI-2 for both samples, but only for the U.S. mothers on the CTSPC, which captured their actual PCA use. Whereas low frustration tolerance was related to Peruvian mothers’ greater abuse risk on both the AAPI-2 and CTSPC, neither greater empathy nor social support satisfaction was predictive of their abuse risk when all of these predictors were considered simultaneously. In contrast, for U.S. mothers, lower frustration tolerance was only predictive of greater actual PCA use on the CTSPC and lower empathy was predictive of higher abuse risk on both the CTSPC and AAPI-2, with no effects observed for social support satisfaction.

Tests of the group differences between U.S. mothers and Peruvian mothers indicated that the two groups differed significantly on two specific paths for the AAPI-2. The Peruvian mothers’ lower frustration tolerance was more significantly predictive of abuse risk on the AAPI-2 than U.S. mothers’ frustration tolerance (Wald (1) = 4.36, *p* < 0.05). In contrast, U.S. mothers’ lower empathy scores more strongly predicted greater abuse risk on the AAPI-2 than that observed for Peruvian mothers (Wald (1) = 7.87, *p* < 0.01). Although the two groups of mothers appeared to differ in their relations between the CTSPC and PCA acceptability, these path coefficients were not statistically different (*p* > 0.05)

## 4. Discussion

The current investigation compared mothers in Peru versus the U.S. to examine potential differences in Social Information Processing (SIP) theory factors (PCA acceptability, frustration tolerance, empathy) and social support in relation to maternal child abuse risk and the use of physical and psychological parent–child aggression (PCA). Although most of the existing research on SIP factors has implied that these factors may be universal, the current findings provide preliminary evidence of potential cultural differences between mothers in the U.S. (where most of the research on SIP regarding child abuse risk has been conducted) and mothers in Peru, suggesting that SIP processes could demonstrate different emphases in SIP processes for different cultural groups. Specifically, whereas the approval of PCA might be more comparable between cultures as a predictor of child abuse risk, Peruvian maternal child abuse risk was more strongly and consistently related to poor frustration tolerance, but U.S. maternal child abuse risk was more strongly and consistently related to lower empathic abilities. Despite some evidence that social support satisfaction was associated with abuse risk for Peruvian mothers, in neither sample was this beneficial resource related to lower child abuse risk upon simultaneously considering the socio-cognitive risk factors of the SIP model.

Utilizing well-controlled statistical models, the current analysis was able to identify the most significant predictors for each group while adjusting for covariance across the predictors and outcome measures. Existing evidence examining SIP theory in child abuse risk has focused largely on U.S. samples [20]. Although the two groups of mothers did not differ in their frustration tolerance scores, low frustration tolerance was consistently associated with greater abuse risk for Peruvian mothers, whereas the effect of this particular SIP factor was apparent for U.S. mothers only in relation to the CTSPC—namely, actual physical and psychological PCA use. These findings are consistent with prior work on the importance of such over-reactivity and frustration in elevating parental abuse risk [37,38]. In contrast, the effect of low empathy was evident solely for the U.S. mothers’ abuse risk (who tended to report more overall empathic ability relative to Peruvian mothers). Although some of the existing research supporting the link between low parental empathy and greater abuse risk has been conducted in Spain [24,35], the role of lower empathy appeared to be less clear, at least in the South American context of Peru. Because low empathy and frustration are intercorrelated, it appears that for Peruvian mothers, low frustration tolerance may be a more salient SIP factor whereas for U.S. mothers, low empathic abilities may be more salient. By examining such factors simultaneously, one may better identify contributors to abuse risk, with current findings suggesting an emphasis of SIP processes that could differ between cultural groups. For example, the current cross-cultural findings underscore the importance of including empathy training for U.S. mothers, but such training may be potentially less relevant for Peruvian mothers. Alternatively, Peruvian mothers may underestimate their empathic abilities, consistent with the cultural values of humility in self-presentation relative to those in collective cultures [73].

One of the critical pre-existing SIP schemas theorized to serve as a precursor for child abuse—approval of PCA—differed between samples. Peruvian mothers reported significantly lower acceptability of PCA than the U.S. mothers, perhaps reflecting the ban on PCA in Peru [56]. Given the legal ban, Peruvian mothers may recognize that PCA is no longer considered acceptable in their society and have begun adopting this social norm compared to its continuing strong approval in the U.S. [54]. However, Peruvian mothers also reported significantly greater use of psychological and physical PCA, relative to U.S. mothers, despite the legal prohibitions in Peru and their own apparent disapproval. Approval of PCA was an abuse risk factor evident for U.S. mothers in the current investigation, an effect that has been demonstrated across multiple countries [22,28,31,32]. However, this effect was observed only on the AAPI-2 abuse risk measure for Peruvian mothers, and not the measure of their actual PCA use. The AAPI-2, in particular, focuses on harsh parenting attitudes and may be particularly inclined to tap this element of child abuse risk [63]. Given all participants self-reported their PCA use, it is possible that either the Peruvian mothers were more willing to disclose their actual use than their U.S. counterparts, or that the Peruvian mothers may engage in behavior about which they may personally disapprove. Beliefs often do not translate into behavior. Such subtle cultural differences from these preliminary findings underscore why more research needs to heed the call of empirically testing the extant research findings outside North America [47,61]. For example, the implications could be that intervention programs for Peruvian mothers may not effectively reduce mothers’ actual PCA use simply by modifying their attitudes approving its use because they may engage in behavior about which they disapprove.

Consistent with a framework that considers both risks and resources, we considered social support as a protective factor independent of the socio-cognitive SIP risk factors. Although prior research has considered social support in relation to child abuse risk, generally finding support for its beneficial effects [39,40,74] (with few exceptions observed, as in South Africa [43]), the current findings surprisingly do not support the role of social support as a resource associated with a lower child abuse risk for either sample. We had speculated that social support would be most relevant for Peruvian mothers given their more collectivistic, interdependent culture [58,75], but Peruvian mothers reported less social support satisfaction than their U.S. counterparts. Conceivably, U.S. mothers may overestimate both their empathic abilities and social support (consistent with more individualist cultural self-presentation styles [73]) relative to Peruvian mothers, again, potentially because of the cultural norms in collectivistic societies to present oneself humbly. Greater social support was significantly associated with lower scores on the AAPI-2 abuse risk measure for Peruvian mothers, consistent with earlier research, but this effect disappeared when considering it simultaneously with the SIP socio-cognitive risk factors, which simply exerted stronger effects. Future cross-cultural work should similarly incorporate both the potential risks and resources collectively with larger samples, in order to more clearly parse their unique contributions, because social support may not be a consistent or adequate resource.

Several limitations in this investigation are worth noting. Foremost among these is the cross-sectional nature of the research design, which prohibits causal interpretations of the SIP factors, although prior longitudinal work suggests that these SIP factors at least predate child abuse risk [22,76]. Additionally, this investigation focused exclusively on mothers, similar to the majority of research on parenting and child abuse, highlighting that cross-cultural research with fathers continues to need urgent attention. The current study also engaged mothers from a single urban center in Peru and one in the southeast U.S.; future work with nationally representative samples would provide greater cross-cultural insights. The samples differed in age, although we statistically controlled for such effects and SIP theory has been applied to parents with children of various ages [see [20] for review]; future cross-cultural work should consider expressly matching samples on multiple demographic characteristics in a case-control design in an effort to better disentangle potential cross-cultural influences beyond the use of statistical controls. Furthermore, the current study relied on self-reports for all constructs; although this might not be an issue for ones’ perceived social satisfaction, foreseeably, social desirability could affect the reporting of one’s approval of PCA, their empathy and frustration tolerance, and their child abuse risk and PCA use. Indeed, as noted above, cultural norms about humility may have led Peruvian mothers to underestimate their own empathic abilities and social support satisfaction. Strategies to include alternative methodologies such as behavioral analog tasks (e.g., [77]) or direct observations of parent–child interactions (see [78]) could prove useful adjuncts in future multimethod research designs. Finally, other risks and resources relevant to child abuse risk should be investigated as we focused on those administered in common between these two samples; notably, fewer contributors were identified for Peruvian mothers, suggesting that additional unmeasured factors may affect Peruvian mothers’ child abuse risk and PCA use.

Given the adverse impact of child abuse [2,3], a greater understanding of the elements that increase the parents’ risk to maltreat their children is sorely needed. Cross-cultural work documents that parenting is strongly influenced by culture [44,48], but research continues to be dominated by work in North America [47,61]. This geographic focus is evident in research on child maltreatment in particular [19], despite the reality that it is clearly a worldwide phenomenon [46]. Identifying the risk and protective factors that may be either universal versus culturally specific can better guide the development of more tailored culturally informed intervention programs that could avert maltreatment wherever it transpires.

## Figures and Tables

**Table 1:** Means and standard deviations by group.

	Peru	U.S.		
	*M* (*SD*/*SE*)	*M* (*SD*/*SE*)	*t*-Test	*p*-Value
Vignette Abuse Definition Total	6.85 (1.41/0.14)	5.58 (1.63/0.12)	6.42	<0.001
Vignette Abuse Severity Total	26.77 (4.45/0.46)	23.83 (4.08/0.30)	5.49	<0.001
Vignette Abuse Reporting Total	6.38 (1.63/0.17)	4.74 (1.63/0.12)	7.97	<0.001
Frustration Discomfort Scale	19.09 (6.03/0.62)	17.97 (6.51/0.49)	1.39	0.165
IRI Combined Empathy Total	50.04 (7.71/0.78)	55.47 (8.35/0.62)	−5.33	<0.001
Social Support Satisfaction	30.04 (6.91/0.71)	40.16 (8.23/0.62)	−10.18	<0.001
AAPI-2 Total	104.48 (20.87/2.11)	98.77 (22.20/1.66)	2.09	0.038
CTSPC Combined Total	31.63 (59.07/6.06)	14.32 (21.82/1.64)	3.48	0.001

*Note*. IRI = Interpersonal Reactivity Index; AAPI-2 = Adult-Adolescent Parenting Inventory-2; CTSPC = Parent–Child Conflict Tactics Scale, Combined Assault. All *p*-values are two-tailed. *SD* = standard deviation; *SE* = standard error.

**Table 2:** Correlations among outcome measures by group.

	1.	2.	3.	4.	5.	6.
1. PCA Acceptability		−0.12	0.13	0.07	−0.34 ***	−0.26 ***
2. Frustration	−0.13		−0.35 ***	−0.14	0.17 *	0.17 *
3. IRI Empathy	0.09	−0.26 **		0.19 *	−0.37 ***	−0.11
4. Social Satisfaction	0.15	−0.10	0.05		−0.11	−0.10
5. AAPI-2 Total	−0.30 **	0.44 ***	−0.14	−0.25 *		0.22 **
6. CTSPC	−0.05	0.16	−0.11	−0.10	0.01	

*Note*. Peruvian sample below the diagonal; U.S. sample above the diagonal. 1. PCA Acceptability = Composite of standardized scores on Vignette Definition of Abuse, Severity, and Reporting subscale total scores; 2. Frustration = Frustration Discomfort Scale total score; 3. IRI = Interpersonal Reactivity Index; 4. Social Satisfaction = Social Support Resources Index, social satisfaction score; 5. AAPI-2 = Adult-Adolescent Parenting Inventory-2; 6. CTSPC = Parent–Child Conflict Tactics Scale (CTSPC) Combined Assault (Physical Assault + Psychological Aggression combined). * *p* < 0.05, ** *p* < 0.01, *** *p* < 0.001.

**Table 3:** Standardized path coefficients by sample.

	Peru		US	
	ß	*p*	ß	*p*
PCA Acceptability → AAPI-2	**−0.22**	**0.003**	**−0.27**	**0.000**
Frustration Tolerance → AAPI-2 *	**0.32**	**0.000**	0.07	0.325
Empathy → AAPI-2 *	0.06	0.471	**−0.23**	**0.000**
Social Support Satisfaction → AAPI-2	−0.11	0.104	0.05	0.395
				
PCA Acceptability → CTSPC	−0.02	0.864	**−0.25**	**0.000**
Frustration Tolerance → CTSPC	**0.24**	**0.020**	**0.21**	**0.044**
Empathy → CTSPC	−0.16	0.134	**−0.23**	**0.001**
Social Support Satisfaction → CTSPC	−0.12	0.178	−0.08	0.347

*Note*: AAPI-2 = Adult-Adolescent Parenting Inventory-2; CTSPC = Parent–Child Conflict Tactics Scale. Mplus models include covariates of age and socioeconomic status. Statistically significant estimates are in bold. Pathways with an asterisk indicate statistically significant differences in the paths between the Peruvian and U.S. mothers.

## Data Availability

The data analyzed in this study are available upon request from the corresponding author. The data are not publicly available in accordance with the consent provided by the participants, and as approved by the University of Alabama at Birmingham Institutional Review Board.
